# Microvascular response to exercise varies along the length of the tibialis anterior muscle

**DOI:** 10.1002/nbm.4796

**Published:** 2022-07-25

**Authors:** Thom T. J. Veeger, Lydiane Hirschler, Celine Baligand, Suzanne L. Franklin, Andrew G. Webb, Jurriaan H. de Groot, Matthias J. P. van Osch, Hermien E. Kan

**Affiliations:** ^1^ C. J. Gorter MRI Center, Dept. of Radiology Leiden University Medical Center (LUMC) Leiden the Netherlands; ^2^ CEA, CNRS, MIRCen, Laboratoire des Maladies Neurodégénératives Université Paris‐Saclay Fontenay‐aux‐Roses France; ^3^ Center for Image Sciences University Medical Centre Utrecht Utrecht the Netherlands; ^4^ Department of Rehabilitation Medicine LUMC Leiden the Netherlands; ^5^ Leiden Institute for Brain and Cognition Leiden University Leiden the Netherlands; ^6^ Duchenne Center the Netherlands

**Keywords:** arterial spin labeling, arterial transit time, exercise, multislice, muscle, perfusion, quantitative

## Abstract

Microvascular function is an important component in the physiology of muscle. One of the major parameters, blood perfusion, can be measured noninvasively and quantitatively by arterial spin labeling (ASL) MRI. Most studies using ASL in muscle have only reported data from a single slice, thereby assuming that muscle perfusion is homogeneous within muscle, whereas recent literature has reported proximodistal differences in oxidative capacity and perfusion. Here, we acquired pulsed ASL data in 12 healthy volunteers after dorsiflexion exercise in two slices separated distally by 7 cm. We combined this with a Look‐Locker scheme to acquire images at multiple postlabeling delays (PLDs) and with a multiecho readout to measure T_2_*. This enabled the simultaneous evaluation of quantitative muscle blood flow (MBF), arterial transit time (ATT), and T_2_* relaxation time in the tibialis anterior muscle during recovery. Using repeated measures analyses of variance we tested the effect of time, slice location, and their interaction on MBF, ATT, and T_2_*. Our results showed a significant difference as a function of time postexercise for all three parameters (MBF: F = 34.0, *p* < .0001; T_2_*: F = 73.7, *p* < .0001; ATT: F = 13.6, *p* < .001) and no average differences between slices over the total time postexercise were observed. The interaction effect between time postexercise and slice location was significant for MBF and T_2_* (F = 5.5, *p* = 0.02, F = 6.1, *p* = 0.02, respectively), but not for ATT (F = 2.2, *p* = .16). The proximal slice showed a higher MBF and a lower ATT than the distal slice during the first 2 min of recovery, and T_2_* showed a delayed response in the distal slice. These results imply a higher perfusion and faster microvascular response to exercise in the proximal slice, in line with previous literature. Moreover, the differences in ATT indicate that it is difficult to correctly determine perfusion based on a single PLD as is commonly performed in the muscle literature.

## Graphical abstract

We used quantitative arterial spin labeling to assess microvascular response to exercise at two locations of the tibialis anterior (TA) muscle. We found initial increased muscle blood flow (MBF) and slower microvascular response to exercise proximally in the TA, indicating regional differences. Arterial transit time (ATT) was decreased after exercise and different between slices. This indicates that commonly used single postlabeling delay measurements have a risk of underestimating perfusion or finding perfusion differences influenced by changes in ATT.

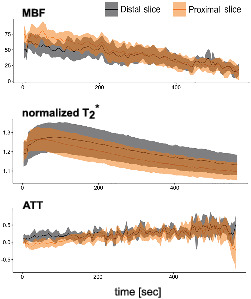

**Abbreviations**
ASL: arterial spin labeling
ATT: arterial transit time
BOLD: blood oxygen level‐dependent
FAIR: flow‐sensitive alternating inversion recovery
FOCI: frequency offset‐corrected inversion
IVIM: intravoxel incoherent motion
MBF: muscle blood flow
M_unsub_
: unsubtracted image
MVC: maximum voluntary contraction
PLD: postlabeling delay
QUIPSS: quantitative imaging of perfusion using a single subtraction
ROI: region of interest
SNR: signal‐to‐noise ratio
SPIR: spectral presaturation with inversion recovery
TA: tibialis anterior
WET: water suppression enhanced through T_1_ effects

## 1 INTRODUCTION

Normal microvascular function is essential for healthy muscles and therefore of interest in studies of muscle physiology and pathology. Microvascular function, in terms of blood perfusion, can be measured using MRI, using either invasive techniques including dynamic contrast‐enhanced MRI, or noninvasive techniques such as intravoxel incoherent motion (IVIM), blood oxygen level‐dependent (BOLD), and arterial spin labeling (ASL). Of these latter three, IVIM and BOLD do not directly measure perfusion. IVIM estimates perfusion‐related measures based on spin dephasing and signal loss, whereas BOLD is a composite measure influenced by more than only perfusion. Therefore, they only provide an indirect and nonquantitative measure of perfusion, whereas ASL directly and quantitatively measures perfusion. However, ASL has a lower signal‐to‐noise ratio (SNR) compared with IVIM and BOLD, and because muscle has a low perfusion at rest compared with brain tissue,^1^, ^2^ this has limited the widespread use of ASL in the field of muscle MRI. In studies of microvascular response to exercise, this limitation is somewhat compensated by an increase in perfusion as a result of the exercise task.

Most studies using ASL in muscle have only reported data from a single slice with the implicit assumption that muscle perfusion is homogeneous.^1^, ^3^, ^4^, ^5^, ^6^ Recent literature, however, has reported proximodistal differences in perfusion‐related parameters and oxidative capacity, measured by IVIM, T_2_*, and ^31^P MRS.^7^, ^8^ Proximodistal differences could be relevant for pathological situations, such as diabetes and peripheral artery disease,^9^, ^10^ because it can be argued that muscle regions with inherently lower perfusion or slower microvascular response to exercise are more vulnerable when perfusion is impaired as result of a disease. Therefore, covering larger imaging volumes is of great interest when assessing microvascular response to exercise.

A recent study acquired multislice pulsed ASL using the flow‐sensitive alternating inversion recovery (FAIR) technique.^11^ In this study, the label was created outside the stack of slices and a correction was needed to account for differences in label arrival time and T_1_ recovery between the slices. However, this technique limits the gap between the slices, thereby limiting muscle coverage. In previous work, we have overcome this limitation by adapting the FAIR^12^ sequence in such a way that the label is also created in between two slices separated by a relatively large distance.^13^ This new split‐label FAIR method enables the assessment of perfusion in two widely separated slices without the need for additional corrections.

Another limitation of previous techniques is that ASL data are acquired at a single postlabeling delay (PLD), with the assumption that the arterial transit time (ATT), that is, the time delay between labeling and the start of label inflow into muscle tissue, is similar between subjects and slices. However, especially in postexercise muscle studies, ATT is most likely not constant over time and postexercise changes in ATT could also be different between participants and/or slices. This is important because Conlin et al.^14^ have shown that the measured perfusion values are highly dependent on the ATT. This can partly be overcome by the use of a “quantitative imaging of perfusion using a single subtraction” (QUIPSS)^15^ module to achieve a sharp labeling bolus. However, the QUIPSS module must be applied shortly after labeling to ensure that the bolus has not passed completely, which limits the SNR and is particularly disadvantageous in muscle tissue.

In the current study, we used the split‐label FAIR^13^ method to quantitatively assess differences in muscle microvascular response to a standardized exercise between two separated slices covering the lower leg. We combined this with a Look‐Locker scheme to acquire images at multiple PLDs and with a multiecho readout to measure BOLD information. This enabled the assessment of microvascular response to exercise using a wide set of parameters. First, quantitative perfusion in terms of muscle blood flow (MBF), the blood supply to the muscle tissue, indicates the amount of muscle perfusion in response to an increase in demand. Second, the BOLD or T_2_* response was measured and is also an indicator for changes in blood flow, but is a composite measure and depends on many other physiological processes such as blood oxygenation, pH, and changes in CO_2_ levels. Lastly, ATT gives an indication of the blood flow velocity and thus the ability to quickly deliver blood to the tissue.

## 2 METHODS

### 2.1 Study participants

Twelve subjects were recruited from a volunteer database without specific inclusion or exclusion criteria other than age of 18–65 years and the absence of MRI contraindications. This study was part of an MRI development protocol in line with local regulations of the medical ethical committee and all participants gave written informed consent prior to participation.

### 2.2 Exercise paradigm

**FIGURE 1:**
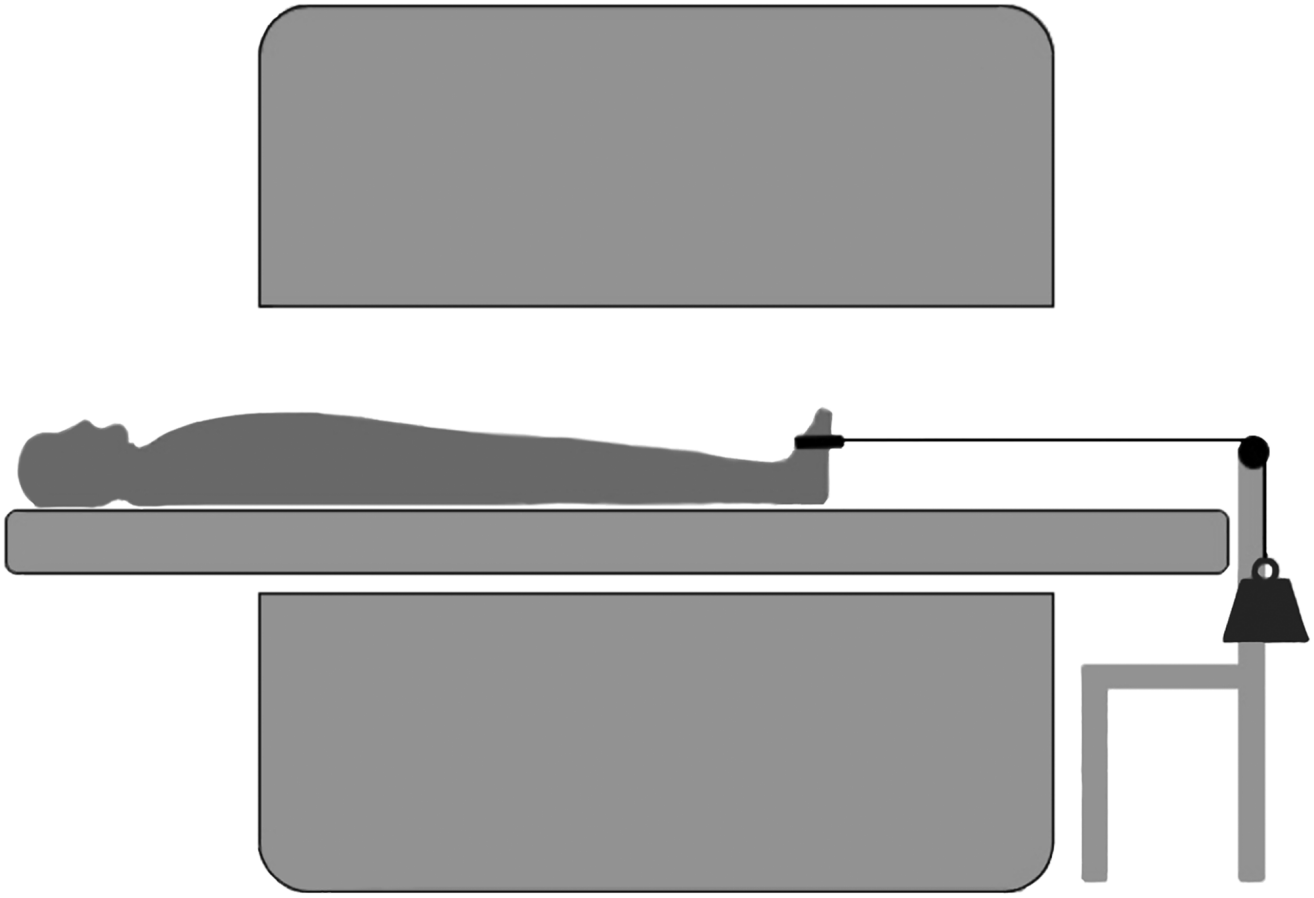
Schematic representation of patient positioning and exercise setup. The load was attached to the foot, just proximal to the toes, using a custom‐built device. The load was determined outside of the MRI and was set to 25% of their maximum voluntary contraction force.

Each volunteer was scanned twice using the split‐label ASL sequence, once during a rest period of 3 min 12 s (resulting in 30 pairs of images), and once both during and after exercise (15 min 12 s, resulting in 150 pairs of images). Approximately 50 pairs of images were acquired during exercise and 100 pairs after exercise. Data during exercise were not included in the analysis because of movement artifacts, but this enabled the shortest time delay after the end of exercise. For the same reason, a researcher stayed inside the scanning room during and after exercise to detach the load without stopping the scanning protocol. The exercise consisted of a 5‐min dynamic dorsiflexion exercise, with a workload set to 25% of the maximum voluntary contraction force (MVC) specifically determined for each individual. MVC was determined before scanning outside of the exercise setup using a handheld dynamometer (MicroFET2; Hoggan Scientific, Salt Lake City, UT, USA). Participants were asked to perform a maximal contraction against the dynamometer and repeated this three times. If the force kept increasing or if the difference between measurements was more than 10%, up to two extra contractions were performed. The largest force recorded during these trials was considered to be the MVC. The personalized load (25% of the individual's MVC) was attached to the foot, just proximal to the toes, using a custom‐built device (Figure 1).

### 2.3 Split‐label ASL scheme

**FIGURE 2:**
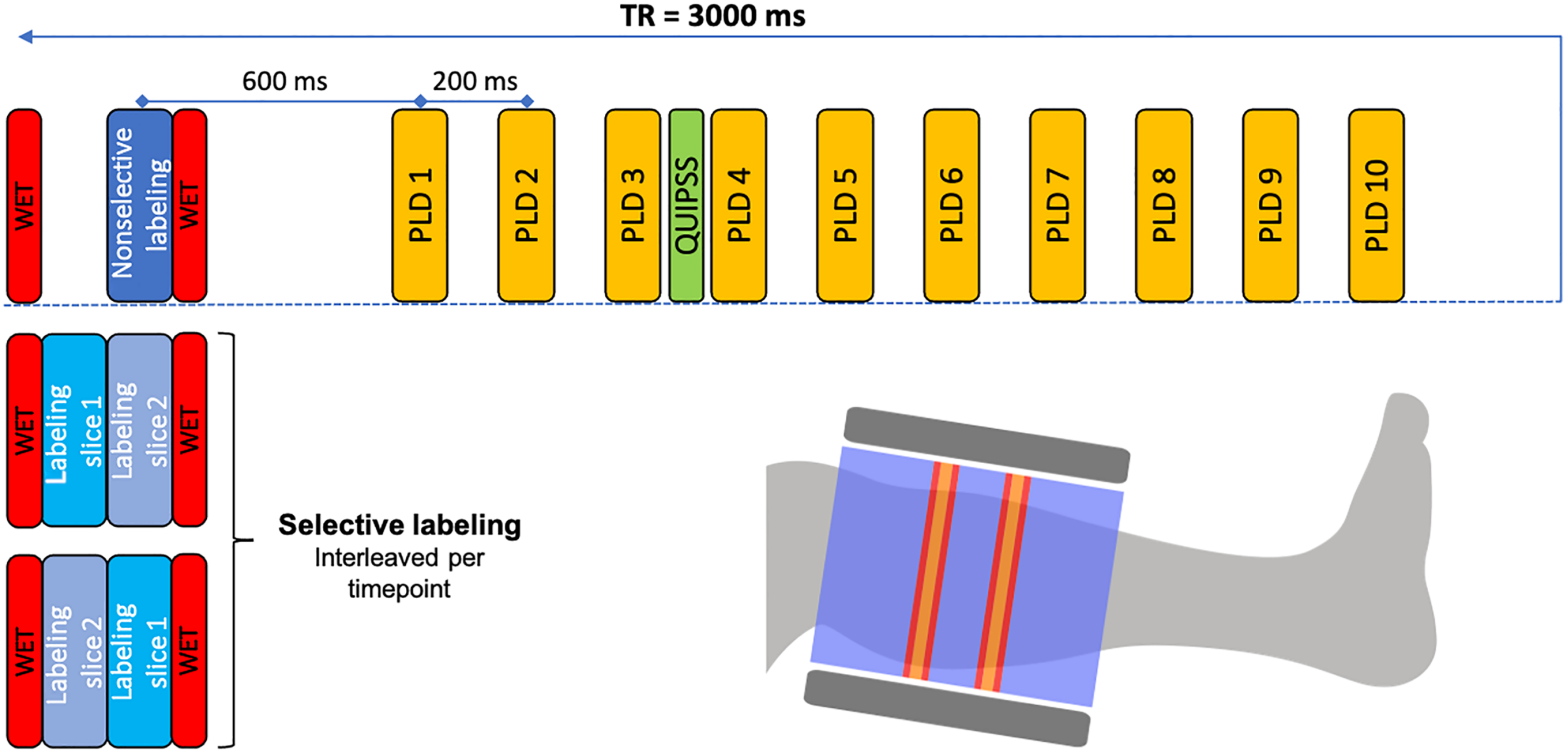
Schematic representation of the labeling scheme. The top row shows the scheme for the nonselective image. For the selective labeling schemes only the first parts are shown in the second and third rows, as the rest is identical to the nonselective labeling scheme. These are interleaved per timepoint (a set of selective and nonselective images) and are switching the order in which the two slices are inverted. Every “PLD” block consists of a SPIR module, an excitation pulse, and a multiband single shot EPI readout. Additionally, a schematic representation of positioning of the imaging slice (orange), presaturation and postsaturation (red) and label (blue) is shown. The position of the coil is indicated in gray. PLD, postlabeling delay; QUIPSS, quantitative imaging of perfusion using a single subtraction; SPIR, spectral presaturation with inversion recovery; WET, water suppression enhanced through T_1_ effects.

The split‐label pulsed FAIR ASL scan, based on Baligand et al.,^13^ was used to assess muscle perfusion. The labeling scheme consisted of presaturation of the imaging slice, followed by either a slice‐selective or nonselective frequency offset‐corrected inversion (FOCI)^16^ pulse of 15‐ms duration, after which postsaturation of the imaging slice was applied. Both the presaturation and postsaturation consisted of a water suppression enhanced through T_1_ effects (WET) module.^17^ To correct for slice‐profile imperfections, the selective inversion slabs were chosen to invert an additional 3 mm on both sides of the imaging slice; for the same reason the presaturation and postsaturation comprised an additional 5 mm on both sides. To achieve labeling in between the two imaging slices, the presaturation and postsaturation and selective inversion were applied selectively for both slices consecutively. For the selective inversion, the order in which the two slices were labeled was interleaved over the timepoints (a pair of selective and nonselective images), to compensate for possible systematic differences between the two slices due to small discrepancies in the timing of the labeling. To make sure that the magnetization transfer effects were the same for both selective and nonselective inversions, the power of the two selective FOCI pulses was halved compared with the non‐selective pulse. A schematic representation of the labeling scheme is shown in Figure 2.

### 2.4 MRI acquisition

All data were acquired on a 3‐T MRI scanner (Ingenia; Philips Healthcare, Best, the Netherlands) using a two‐channel body transmit coil and an eight‐element small extremity flexible receive array placed around the right lower leg. The participants were positioned supine and feet first in the scanner. The full protocol consisted of two types of sequences, in addition to the survey scans, and took 21 min 14 s in total:

1. A three‐point multiacquisition Dixon scan for anatomical reference (TR/TE/∆TE 400/4.41/0.76 ms; flip angle 8°; FOV 190 x 190 x 86 mm^3^; voxel size 1.5 x 1.5 x 8 mm^3^; slice gap 70 mm; two transverse slices). The center between the two slices was positioned 40% of the length of the tibia bone from the tibial head and perpendicular to the tibia bone.
2. The split‐label ASL scan was performed at the same location as the Dixon images. A single‐shot three‐echo EPI was acquired to assess muscle perfusion and T_2_* relaxation time with a single sequence (TR/TE/∆TE 3000/14.0/17.4 ms; SENSE factor 2.3; FOV 190 x 190 x 86 mm^3^; voxel size 3 x 3 x 8 mm^3^; slice gap 70 mm; two transverse slices). In addition, a Look‐Locker scheme with variable flip angle was used to acquire signal at 10 PLDs ranging from 600 to 2400 ms and evenly spaced in time to enable MBF and ATT quantification. The variable flip angles were chosen in such a way that the theoretical magnetization after excitation was comparable between the Look‐Locker phases and were set to 19°, 20°, 21°, 23°, 25°, 27°, 31°, 36°, 45°, and 90°. Both slices were acquired simultaneously using a vendor‐supplied multiband approach. To allow for signal quantification, a QUIPSS^15^ module was applied proximal to both slices in the time between the third and fourth PLD to obtain a sharp labeling bolus after 1000 ms.^18^ It consisted of two 44‐mm saturation slabs applied 10 mm proximal to the imaging plane. Spectral presaturation with inversion recovery (SPIR) was used to suppress fat signal originating from the subcutaneous fat and bone marrow.

### 2.5 Data analysis and quantification

Data analysis was performed in MATLAB (R2019b; MathWorks, Natick, MA, USA). Postexercise timepoints contaminated by subtraction errors as a result of movement were identified and excluded from analysis. These were identified by calculating the averaged ASL signal (selective – nonselective) from the first echo of the sixth PLD over the complete image acquired at each timepoint and by performing an outlier analysis using the built‐in MATLAB function *isoutlier*, selecting datapoints more than three scaled median absolute deviations away from the median. Thereafter, the ASL and unsubtracted signal were corrected for the effects of the variable flip angles applied during the Look Locker readout by dividing each scan by the sine of the flip angle and correcting for the loss of label due to the preceding RF pulses.

#### 2.5.1 Data postprocessing

For every PLD and timepoint, voxel‐wise *ASL* maps were calculated from the first echo using a surround subtraction scheme^19^ by interleaving

(1a) $$\mathrm{ASL}=\frac{S_{i}+S_{i+1}}{2}-{\mathrm{NS}}_{i}$$

with

(1b) $$\mathrm{ASL}=S_{i+1}-\frac{{\mathrm{NS}}_{i}+{\mathrm{NS}}_{i+1}}{2},$$

where S and NS are selective and nonselective images, respectively, and i is the timepoint before subtraction. One pair of *ASL* images was acquired with a time resolution of 6 s; after surround subtraction this resulted in a reconstructed time resolution of ~3 s.

In order to have the same time resolution for the unsubtracted images ( $M_{\text{unsub}}$), used for *T*_2_* and *M*_0_ quantification, a surround averaging was used by interleaving

(2a) $$M_{\text{unsub}}=\frac{\frac{S_{i}+S_{i+1}}{2}+{\mathrm{NS}}_{i}}{2}$$

with

(2b) $$M_{\text{unsub}}=\frac{S_{i+1}+\frac{{\mathrm{NS}}_{i}+NS_{i+1}}{2}}{2}.$$

#### 2.5.2 Regions of interest

Regions of interest (ROIs) enclosing the tibialis anterior (TA) muscle were manually drawn on the scanner‐reconstructed water Dixon image. The ROIs were drawn just within the fascia and the intramuscular tendon was excluded. The water Dixon images were registered to all acquired timepoints of the selective and nonselective images using the built‐in MATLAB function *imregdemons* and the resulting displacement fields were used to deform the ROI to every acquired timepoint. To obtain an ROI for every timepoint after surround subtraction (which uses three images), only voxels included in all three ROIs used for that timepoint were included.

Next, a blood vessel mask was created by calculating an average rest ASL image, where voxels with ASL signal more than two standard deviations above average were assumed to contain signal originating from blood vessels. The same procedure was applied to the last 100 ASL timepoints of the exercise scan. Two masks were used, because movement during the exercise in between the two scans would render a single mask susceptible to erroneous inclusion of arterial voxels. These masks were applied to all the at‐rest timepoints and all timepoints after exercise to exclude voxels with arterial signal.

#### 2.5.3 ASL quantification

The average unsubtracted and ASL signal were calculated per ROI, for every slice, PLD, and timepoint. The following equation was fitted to the average M_unsub_ using a least squared method to estimate the *M*_0_ for every timepoint:

(3) $$M_{\text{unsub}}=M_{0}\cdot \left(1-e^{-\mathrm{PLD}/T_{1\text{blood}}}\right),$$

where the T_1blood_ was set to 1.650 s at 3 T.^20^

The average ASL signal per ROI was used for quantification First, a three‐window moving average was applied over all timepoints. After that, for both slices and every timepoint, a least squares method was used to fit MBF and ATT per timepoint according to the Buxton general kinetic model for pulsed ASL^21^:

(4) $$\begin{array}{cc} M_{\mathrm{sel}}-M_{\text{nonsel}}=0 & \;0<\mathrm{PLD}<\mathrm{ATT}\\ \;=2\cdot M_{0}\cdot f\cdot \left(\mathrm{PLD}-\mathrm{ATT}\right)\cdot \alpha \cdot e^{-\mathrm{PLD}/T_{1\text{blood}}}\cdot q_{p}\left(\mathrm{PLD}\right)\; & \;\mathrm{ATT}<\mathrm{PLD}<\tau +\mathrm{ATT}\\ \;=2\cdot M_{0}\cdot f\cdot \tau \cdot \alpha \cdot e^{-\mathrm{PLD}/T_{1\text{blood}}}\cdot q_{p}\left(\mathrm{PLD}\right) & \tau +\mathrm{ATT}<\mathrm{PLD} \end{array}$$

with

$$\begin{array}{cc} q_{p\left(\mathrm{PLD}\right)}=\frac{e^{k\cdot \mathrm{PLD}}\cdot \left(e^{-k\cdot \mathrm{ATT}}-e^{-k\cdot \mathrm{PLD}}\right)}{k\cdot \left(\mathrm{PLD}-\mathrm{ATT}\right)} & \;\mathrm{ATT}<\mathrm{PLD}<\tau +\mathrm{ATT}\\ \;=\frac{e^{k\cdot \mathrm{PLD}}\cdot \left(e^{-k\cdot \mathrm{ATT}}-e^{-k\cdot \left(\tau +\mathrm{ATT}\right)}\right)}{k\cdot \tau }\; & \tau +\mathrm{ATT}<\mathrm{PLD}\\ \;k=\frac{1}{T_{1\text{blood}}}\cdot \frac{1}{T_{1\text{muscle}}}+\frac{\mathrm{MBF}}{\lambda } & \end{array}\;,$$

where M_0_ was the average value after exercise, α was the labeling efficiency and was assumed to be 0.95 for pulsed ASL,^20^ λ was the tissue‐blood partition coefficient and was set to 0.9,^20^ and T_1muscle_ was assumed to be 1.420 s at 3 T.^22^ The values for α and λ were retrieved from brain literature, as these values are not yet known for muscle tissue. The bolus width, τ, was calculated as the time between the nonselective and QUIPSS pulses, with values of 1.087 s for the proximal slice and 1.097 s for the distal slice. *MBF* was calculated by multiplying *f* by 6000 to convert it to units of ml/min/100 g. The MBF and ATT time series were checked for outliers due to any issues in the fit, using the built‐in MATLAB function *isoutlier* and a moving median method with a 50‐timepoint window; outliers were subsequently excluded from further analysis. The SNR of the pre‐exercise rest scan was too low to properly quantify baseline MBF and ATT values and therefore MBF and ATT were not normalized to pre‐exercise baseline.

#### 2.5.4 T_2_* quantification

For calculation of the T_2_* relaxation time, the three echoes from the last PLD of the unsubtracted images were used. T_2_* maps were created by fitting a mono‐exponential decay to the three datapoints using a dictionary method on a voxel‐by‐voxel basis. This dictionary was created using T_2_* values between 0 and 50 ms with steps of 0.05 ms. Voxels that fitted on the boundaries of the dictionary were excluded because they cannot be physiologically correct. The average T_2_* was calculated per ROI for every slice, PLD, and timepoint, and was normalized to the baseline T_2_* calculated from the pre‐exercise rest scan.

### 2.6 Statistical analysis

To test the effect of time postexercise, slice location, and their interaction (time postexercise x slice location) on the MBF, T_2_*, and ATT, three separate repeated measures analyses of variance (ANOVAs) were performed. Each of these analyses included time postexercise, slice location, and their interaction as predictors for MBF, T_2_*, or ATT. Time postexercise was not included as a continuous but as a factorized variable to the analyses to reduce the correlation between the timepoints, thereby avoiding an overestimation of the power. The time series were split into five equal time windows of 113 s and the average within those time windows was used for the analysis.

To visualize the effect of slice location, time postexercise and their interaction for MBF, T_2_*, and ATT in more detail, the difference between the slices (proximal – distal) was calculated at every timepoint for all participants. The average difference and 95% confidence intervals (CIs) were calculated for all timepoints. For the timepoints where the 95% CI did not include zero, the difference between the two slices was considered significant.

Any gaps in the MBF, T_2_*, and ATT time series, due to outlier removal, were filled using a linear interpolation method in MATLAB. The rest of the statistical analyses were conducted using the software package R (R Core Team, 2019) in combination with the package *ez*.^23^ The significance level was set to *p* less than .05, *p* values were corrected with the Greenhouse–Geisser correction if the sphericity assumptions were not met, and additionally all *p* values were adjusted with a false discovery rate correction (Benjamini–Hochberg).^24^

## 3 RESULTS

**FIGURE 3:**
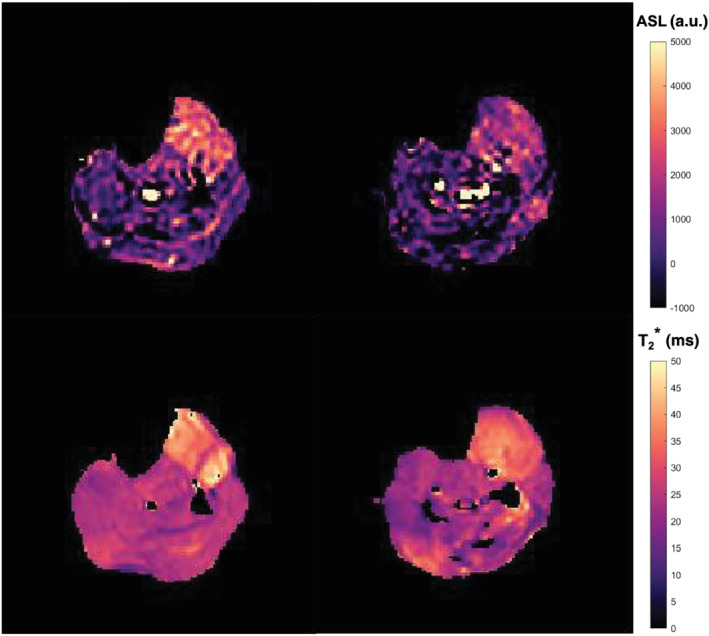
Representative ASL (top) and T_2_* (bottom) maps shortly after exercise for the proximal (left) and distal (right) slice. Within the T_2_* maps, voxels that were excluded because they fitted on the boundaries of the dictionary appear in black. ASL, arterial spin labeling

In total, 12 volunteers (five females and seven males; age = 34 ± 15 years; height = 176.9 ± 10.2 cm) were included in this study. The average exercise load corresponding to 25% MVC was 8.4 ± 1.2 kg. Representative postexercise ASL and T_2_* maps are presented in Figure 3 and showed mainly a signal increase in the anterior compartment of the lower leg, as is expected given the dorsal flexion exercise task.

**FIGURE 4:**
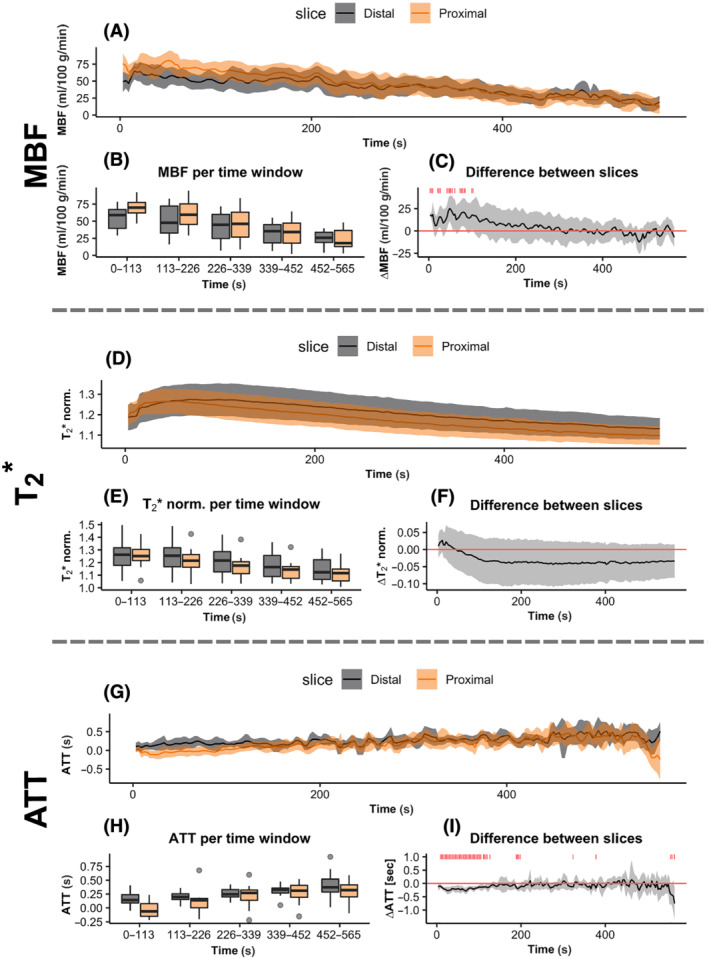
The average postexercise MBF, normalized T_2_*, and ATT as a function of time postexercise averaged over all participants are shown in (A), (D), and (G), respectively. The distal slice is indicated in black and the proximal slice in orange and the bands indicate the 95% confidence intervals (CIs). (B), (E), and (H) show boxplots with the time series split into time windows of 113 s that are used for the repeated measures ANOVAs. The median is indicated by the thick black lines, the lower and upper hinges correspond to the first and third quartile, respectively, and the whiskers extend from the hinge to the value no further than 1.5 times the interquartile range; outlying datapoints outside the whiskers are indicated by gray dots. In (C), (F), and (I), the average difference in MBF, normalized T_2_*, and ATT between the two slices (proximal – distal) over all participants as a function of time postexercise are shown, respectively, with the bands indicating the 95% CIs. On the timepoints where the 95% CIs do not include zero, a red marker is shown above the time series. ANOVAs, analyses of variance; ATT, arterial transit time; MBF, muscle blood flow; norm., normalized to baseline value.

The average MBF and T_2_* time curves after exercise showed a clear increase followed by a recovery back to baseline, while ATT was decreased after exercise before returning to baseline (Figure 4A,B, D,E, and H,I, respectively). The repeated measures ANOVAs yielded significant differences as a function of time postexercise for MBF, T_2_*, and ATT, while no difference between slices was observed for any of the parameters (Table 1; Figure 4B,E,H). The interaction between time postexercise and slice location was significant for MBF and T_2_*, but not for ATT.

**TABLE 1:** Results of repeated measures ANOVAs

MBF	*F value*	*p value*	*Adjusted p value*
Slice location (proximal ‐ distal)	0.84	.378	.378
Time postexercise (s)	33.98	<.0001	**<.0001**
Time postexercise x slice location	5.53	.010	**.018**

T_2_* norm.			
Slice location (proximal ‐ distal)	1.37	.266	.299
Time postexercise (s)	73.70	<.0001	**<.0001**
Time postexercise x slice location	6.05	.008	**.018**

ATT			
Slice location (proximal ‐ distal)	5.06	.046	.069
Time postexercise (s)	13.58	<.0001	**<.001**
Time postexercise x slice location	2.21	.123	.159

*Note*: Adjusted *p* values are calculated using the false discovery rate correction, significant adjusted *p* values are indicated in bold. Abbreviations: ANOVAs, analyses of variance; ATT, arterial transit time; MBF, muscle blood flow; norm., normalized to baseline value.

**FIGURE 5:**
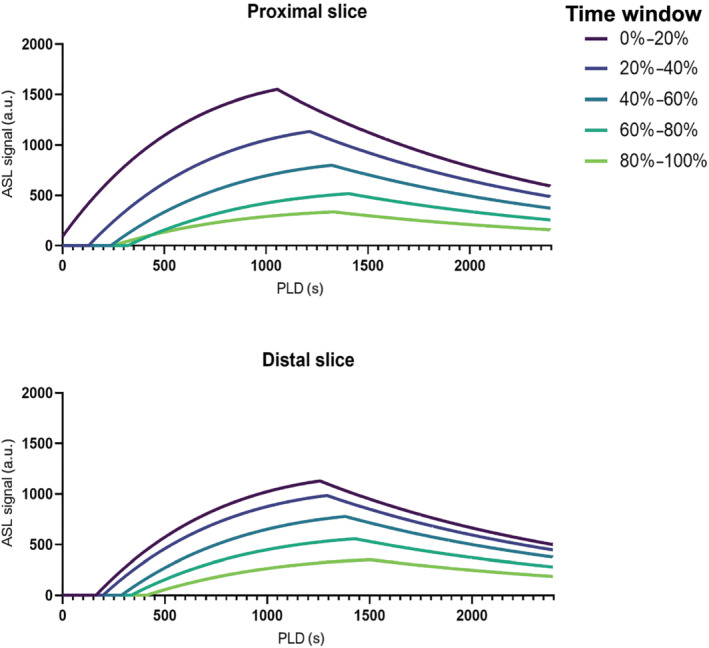
The average Buxton fit for each time window over all participants after exercise for the proximal and distal slice. These curves are reconstructed by using Equation (4) and the average ATT and MBF values over all participants and timepoints to calculate the ASL signal (selective – nonselective) at every PLD between 0 and 2400 ms. ASL, arterial spin labeling; ATT, arterial transit time; MBF, muscle blood flow; PLD, postlabeling delay.

The average difference between the two slices as a function of time postexercise is shown in Figure 4C,F,I and shows the interaction effect between the time postexercise and slice location. Figure 4C,I show that for MBF and ATT, significant differences between the two slices occurred within the first 2 min postexercise, with a higher MBF and a lower ATT for the proximal slice. After about the first 200 s postexercise, the difference between the two slices was negligible. The T_2_* showed a delay between the time curves of the two slices over the full time course postexercise, with the proximal slice preceding the distal slice. From the 95% CIs, it can be seen that the difference itself was highly variable. A representation of the average MBF and ATT over all participants and timepoints within the five time windows is shown in Figure 5. It shows the later inflow of label and lower ATT in the proximal slice, especially just after exercise, and the higher MBF in the proximal slice.

## 4 DISCUSSION

In this study we used split‐label FAIR to quantitatively assess the differences in muscle microvascular response to exercise between two slices separated by 7 cm covering the lower leg. Our results show that there is a significant interaction between time postexercise and slice location effects for MBF and T_2_*. The most prominent difference between the two slices was observed during the first 2 min postexercise, with significantly higher MBF and lower ATT in the proximal slice at multiple timepoints, primarily in the first 200 s postexercise. For T_2_*, the results showed a delayed response in the distal slice compared with the proximal slice, but no absolute difference in T_2_* between the two slices was found. Additionally, a significant effect of time postexercise for ATT was observed, with decreased ATT values after exercise before returning to baseline.

The increased MBF in the proximal slice during the first 2 min postexercise is in good agreement with the literature,^7^, ^8^, ^11^ suggesting a proximodistal gradient in muscle perfusion. Moreover, the significant interaction effects between time postexercise and slice location indicate a faster microvascular response to exercise in the proximal slice, reflected by faster MBF recovery, an earlier peak in T_2_*, and a lower ATT (i.e., shorter arrival times) during the first 2 min postexercise. The difference in muscle perfusion and microvascular response to exercise between two separated slices along the proximodistal axis is in line with data in the literature assessing oxidative capacity in muscle.^7^, ^8^, ^11^ The specific delayed response in T_2_* in the distal slice is in line with the findings reported by Boss et al.^7^ The reason for the proximodistal differences is still under debate. One possible reason could be that the number of oxidative fibers increases from the distal to the proximal end, as was found in rodents.^25^ However, Heskamp et al.^8^ did not find differences in carnosine levels, a biomarker for fiber type, along the TA. Another reason could be that the capillary density is lower distally than proximally, as has also been found in the TA of rats.^26^ Finally, the feeding artery most commonly enters the TA near the proximal end of the muscle,^27^, ^28^ therefore the distance to the distal slice might be larger compared with the proximal slice, which could lead to lower blood flow velocities at the distal slice.

A lower regional perfusion and slower microvascular response to exercise could be of importance for pathologies where the muscle's ability to adequately match the oxygen demand during activity is impaired and where regional differences in disease progression have been observed, such as in Duchenne and Becker muscular dystrophies.^29^, ^30^, ^31^, ^32^, ^33^ One might argue that regions with inherently lower perfusion and exercise response are especially vulnerable to impairments due to a pathology, which might result in a heterogeneous disease progression within the muscle. To assess the clinical implication of regional differences in microvascular response to exercise, it would be interesting to relate these differences to damage because of impairments in oxygen supply.

Our data also enabled the assessment of ATT, which was shown to be shorter just after exercise, increasing over time thereafter, and was shorter in the proximal compared with the distal slice during the first 2 min of recovery. The shorter ATT just after exercise is in line with previous work using dynamic contrast‐enhanced imaging showing that ATT decreases with increasing workload.^14^ More specifically, we found that the ATT was shorter just after exercise, meaning that the labeled blood travels faster to the imaged region. As a result, the peak ASL signal is reached at an earlier PLD compared with rest (Figure 5). With a commonly used PLD value of 1600 ms the MBF just after exercise will be underestimated and will result in lower peak MBF values. Supporting this, previous literature with a relatively long PLD (≥ 1500 ms) reported lower MBF values,^3^, ^5^ while another study with a relatively short PLD (1000 ms) reported similar MBF values compared with ours.^4^ As these differences in ATT make it difficult to correctly quantify MBF based on one single PLD, differences found in studies assessing perfusion using a single PLD acquisition could also be partly dependent on changes in ATT.

The proximodistal differences found in previous papers are in general more pronounced than those found in the current study, although a direct comparison is difficult due to distinct study designs and readouts.^7^, ^8^, ^11^ The relatively large differences found by Heskamp et al.^8^ and Boss et al.^7^ can be explained by a larger coverage (180 vs. 70 mm). Heskamp et al.^8^ only found differences in blood flow‐related parameters between the most distal and most proximal slice and Boss et al.^7^ also found the largest differences between the outer slices. In addition, the proximodistal differences found in these studies suggest a nonlinear gradient, therefore difference between slices might depend on the chosen slice location. Moreover, both used an isometric instead of a dynamic exercise paradigm. Niess et al.^11^ covered a similar region as in the current study but analyzed the calf muscles instead of the TA.

Some limitations of the current study should be mentioned. First, although the exercise was mostly standardized, participants were still able to choose the exercise speed themselves. However, this most likely only affected the differences between participants and had a limited effect on intramuscular differences. Second, the intersubject variability in our data was quite high. Apart from the factors mentioned above, this could be due to the wide range in age and height of the subjects. However, these confounding factors did not significantly correlate to any of the outcome parameters (data not shown). Third, we also found negative ATT values, which by definition is impossible. This can most likely be attributed to the relatively late first PLD of 600 ms. As can be seen in Figure 5, with a PLD of 600 ms, only a small portion of the label inflow is captured and it is therefore difficult to estimate precisely when the inflow of label started. Nevertheless, we expect that this will have resulted in a limited influence on the difference between the two slices. Last, we only acquired data from the right leg and did not account for leg dominance. However, only one of the participants was left dominant, therefore this will most likely have had a negligible effect on the results.

To conclude, we observed significant differences microvascular response to exercise at two positions along the TA's proximodistal axis and showed differences in ATT between two slices postexercise. These results indicate that the commonly used single PLD measurements have a risk of either underestimating perfusion or finding perfusion differences influenced by changes in ATT. Lower regional perfusion and slower microvascular response to exercise can be of special interest for pathological situations, as these regions could well be more vulnerable to impairments as a result of the pathology.
